# From infection to infarction: cytomegalovirus retinitis complicated by retinal ischemia and vitreous hemorrhage in the setting of JAK inhibition - a case report

**DOI:** 10.1186/s12886-025-04558-y

**Published:** 2026-01-12

**Authors:** Ciera D. Johnson, Lulwa El Zein, Justin Yamanuha

**Affiliations:** 1 University of Minnesota Medical School, 420 Delaware St SE, Minneapolis, MN 55455 USA; 2 Department of Ophthalmology and Visual Neurosciences, University of Minnesota, Phillips Wangensteen Building, 9th Floor 516 Delaware St SE, Minneapolis, MN 55455 USA

**Keywords:** Upadacitinib, Janus kinase inhibitors, Cytomegalovirus retinitis, Immune recovery uveitis, Retinal ischemia, Vitreous hemorrhage

## Abstract

**Background:**

Cytomegalovirus (CMV) retinitis has been increasingly reported in patients receiving Janus kinase (JAK) inhibitors, yet vision-threatening ischemic complications in this population remain poorly characterized. Immune recovery uveitis (IRU) and cystoid macular edema (CME) are recognized post-infectious sequelae, but delayed retinal ischemia and hemorrhage in the setting of JAK inhibition are uncommon. We present a case that illustrates these complications in a patient on upadacitinib for rheumatoid arthritis.

**Case presentation:**

An 81-year-old woman with rheumatoid arthritis on upadacitinib for approximately one year and long-term hydroxychloroquine presented with decreased vision and floaters in her left eye. Examination revealed vitritis, focal hemorrhagic retinitis, and an epiretinal membrane without CME. Anterior chamber paracentesis confirmed CMV infection via aqueous humor polymerase chain reaction, guiding prompt antiviral therapy. She received oral valganciclovir and alternating intravitreal ganciclovir and foscarnet, though systemic therapy was later discontinued due to leukopenia. After cessation of upadacitinib and hydroxychloroquine, the retinitis stabilized, but she developed IRU with new-onset CME. Weeks later, she presented with a dense vitreous hemorrhage without retinal tears or detachment. Fluorescein angiography revealed temporal retinal ischemia remote from the original retinitis. Intravitreal bevacizumab led to improvement in both CME and hemorrhage. Pan-retinal photocoagulation is planned to reduce the risk of recurrent hemorrhage.

**Conclusions:**

This case illustrates CMV retinitis in the setting of JAK inhibition complicated by delayed retinal ischemia and vitreous hemorrhage arising remote from the original retinitis. Although ischemic vasculopathy is a recognized complication of CMV retinitis among non-HIV immunosuppressed patients, such sequelae have been infrequently documented in JAK inhibitor-associated cases. Ophthalmologists should maintain a high index of suspicion for CMV retinitis in this emerging population and continue close surveillance for delayed ischemic complications even after apparent disease stabilization.

## Background

Janus kinase (JAK) inhibitors are increasingly used to treat inflammatory conditions, including rheumatoid arthritis (RA), inflammatory bowel disease, and atopic dermatitis [1]. These agents modulate intracellular cytokine signaling and have demonstrated efficacy with generally favorable safety profiles. However, JAK inhibitors are associated with an increased risk of opportunistic infections [2]. Patients receiving JAK inhibitors have higher rates of infections, including herpes zoster, candidiasis, and tuberculosis reactivation, compared to the general population, particularly at higher doses or in combination with other immunosuppressants [2, 3].

Cytomegalovirus (CMV) is a herpesvirus that can remain latent in hematopoietic and endothelial cells and reactivate under conditions of immunosuppression [3]. CMV retinitis is typically associated with hemorrhagic retinitis with a granular pattern, but it may progress to tissue necrosis, retinal detachment, or immune recovery uveitis (IRU), often resulting in vision loss [4, 5].

Reports of CMV retinitis in patients receiving JAK inhibitors remain limited, especially when excluding cases with HIV, transplantation, or malignancy. To the best of our knowledge, only three prior cases have been published: two in patients treated with tofacitinib and one in a patient receiving upadacitinib [6–9]. None of these cases described ischemic complications. Ischemic complications of CMV retinitis, including occlusive vasculitis, nonperfusion, and neovascularization, are well documented in non-HIV immunosuppressed hosts. The case described by Cho et al. demonstrated severe ischemic CMV retinitis in a stem-cell transplant recipient on daratumumab, but not in the setting of JAK inhibition [10]. This distinction highlights that, while ischemia can occur in CMV retinitis, its occurrence in association with JAK inhibitor therapy appears uncommon.

We describe a case of CMV retinitis in an 81-year-old woman receiving upadacitinib, complicated by retinal ischemia and vitreous hemorrhage that developed remote from the original retinitis, an uncommon ischemic pattern among the limited reported cases of JAK inhibitor-associated CMV retinitis. This case emphasizes the need for heightened vigilance for atypical ocular complications in patients on JAK inhibitors and contributes to the growing literature on opportunistic infections in this emerging therapeutic context.

## Case presentation

### Initial presentation

An 81-year-old woman with RA on upadacitinib and long-term hydroxychloroquine, chronic kidney disease, clonal cytopenia of undetermined significance, and hypertension presented with decreased vision and floaters in her left eye. She had no history of HIV infection, organ transplantation, or prior herpes viral infections; she had received recombinant herpes zoster vaccination. Best-corrected visual acuity was 20/25 in the right eye and 20/60 in the left eye. Intraocular pressures were 15 mmHg bilaterally. Extraocular motility was full, pupils were normal without afferent pupillary defects, and an inferonasal visual field defect was noted in the left eye.

**Fig. 1:**
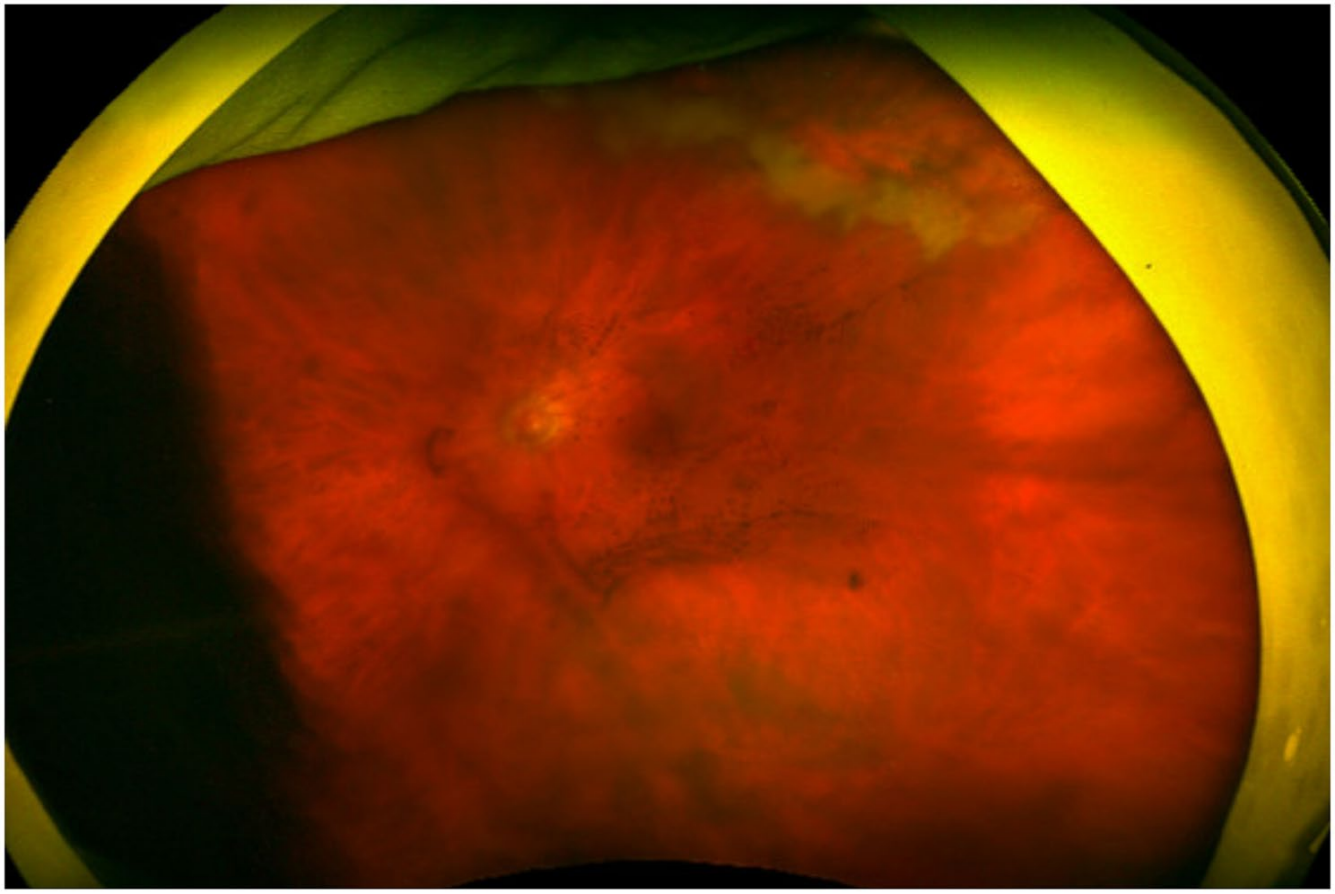
Widefield fundus photo of the left eye demonstrating dense vitritis and a focal line of hemorrhagic retinitis in the superotemporal periphery

Slit lamp examination revealed 2 + anterior chamber cell and 1–2 + flare in the left eye; the right eye was unremarkable. Fundus examination of the left eye showed significant vitritis and a focal line of hemorrhagic retinitis in the superotemporal periphery (Fig. 1). Given her age and moderate immunosuppression on a JAK inhibitor, an opportunistic viral infection was suspected. Anterior chamber paracentesis was performed to obtain aqueous humor for polymerase chain reaction (PCR) testing, which confirmed CMV infection while excluding herpes simplex and varicella-zoster virus. This step was crucial, as CMV is typically observed in severely immunocompromised patients, and early targeted therapy depends on precise viral identification. The aqueous PCR was qualitative rather than quantitative due to institutional testing constraints, and a fluorescein angiogram was not obtained at initial presentation.

### Differential diagnosis

Unilateral vitritis with focal hemorrhagic retinitis in this patient prompted a broad differential, including CMV, herpes simplex virus, varicella-zoster virus, toxoplasmosis, syphilis, and tuberculosis. Non-infectious etiologies such as lupus-related retinitis, sarcoidosis, or Behcet’s disease were considered less likely given the patient’s history of RA. Recognition of CMV in a moderately immunosuppressed patient was a diagnostic challenge, highlighting the need for high clinical suspicion in patients on JAK inhibitors.

### Treatment

The patient received oral valganciclovir 900 mg twice daily and alternating intravitreal injections of ganciclovir (2 mg/0.05 mL) and foscarnet (1.2 mg/0.05 mL) every 2–7 days, for a total of eight ganciclovir and seven foscarnet injections. Due to significant gastrointestinal intolerance, the valganciclovir regimen was reduced to 450 mg three times daily as an individualized dosing strategy to improve tolerability while maintaining systemic antiviral exposure. After consultation with rheumatology, upadacitinib and hydroxychloroquine were discontinued to mitigate ongoing immunosuppression. Approximately two months later, valganciclovir was held due to leukopenia and subsequently discontinued following evaluation by Hematology. The CMV retinitis stabilized even after cessation of intravitreal therapy. Retinitis stability was defined clinically by the absence of active borders at the original lesion and the lack of any new retinal lesions in either eye. Several weeks after stabilization, the patient subsequently developed IRU with new-onset cystoid macular edema (CME), treated with topical steroids and nonsteroidal drops.

### Outcome and follow-up

**Fig. 2:**
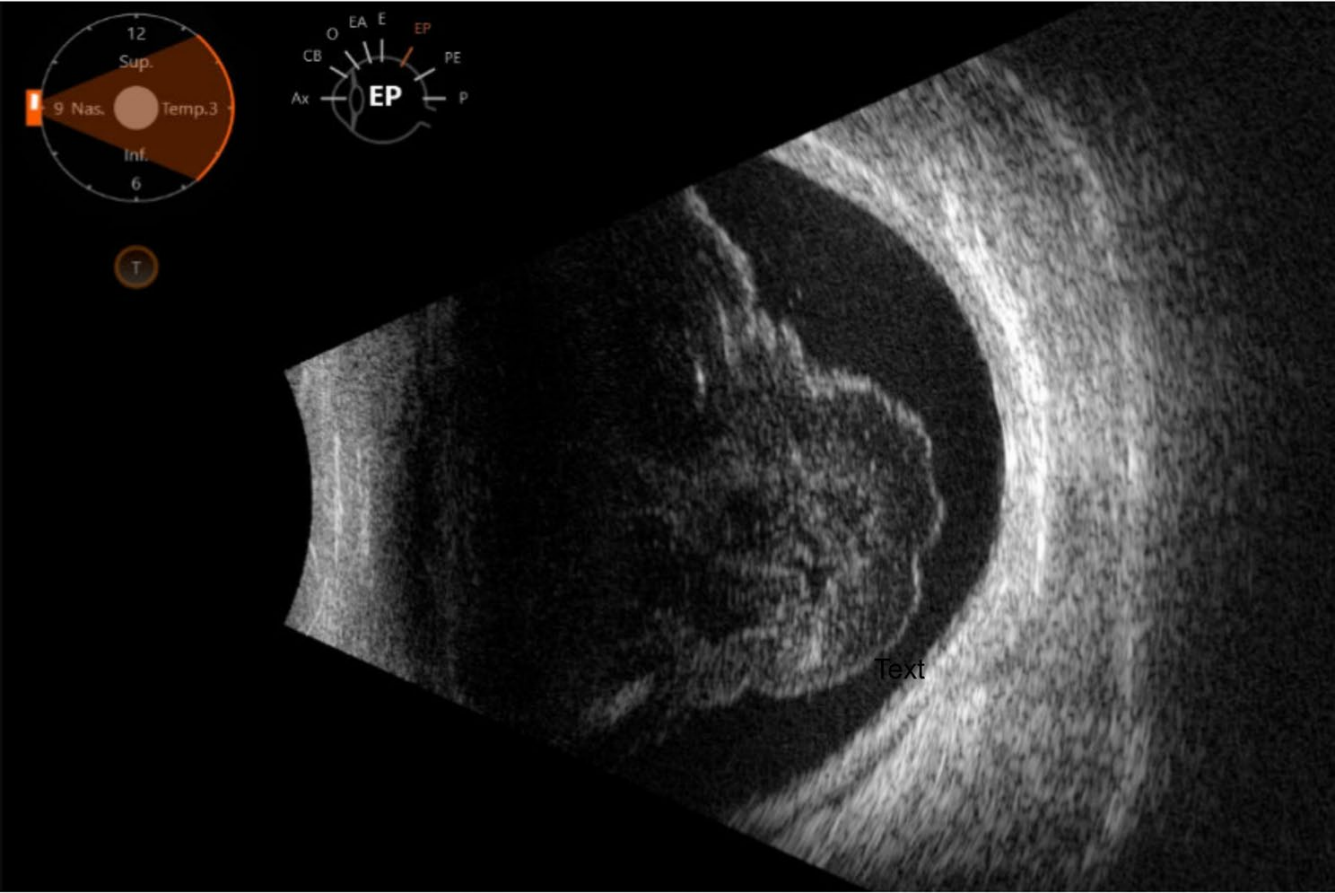
B-scan ultrasound of the left eye demonstrating dense vitreous hemorrhage without retinal tears or detachment

**Fig. 3:**
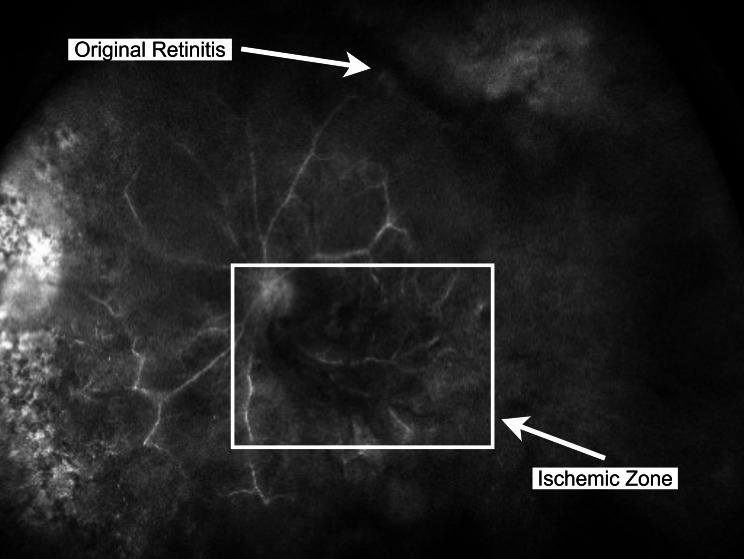
Widefield fluorescein angiogram of the left eye revealing temporal ischemia without retinal neovascularization. The ischemic zone (3–5 o’clock) is anatomically remote from the area of prior CMV retinitis (12 − 2 o’clock)

Weeks after retinitis stabilization and following the onset of IRU, her vision acutely declined to hand movements in the left eye. Examination revealed a dense vitreous hemorrhage without retinal tears or detachment, confirmed by B-scan ultrasound (Fig. 2). Fluorescein angiography identified retinal ischemia in an area distant from the original retinitis (Fig. 3), an atypical distribution that could have been misattributed to age-related vascular disease. This highlighted the diagnostic challenge of recognizing delayed CMV-related ischemic complications remote from prior retinitis.

**Fig. 4:**
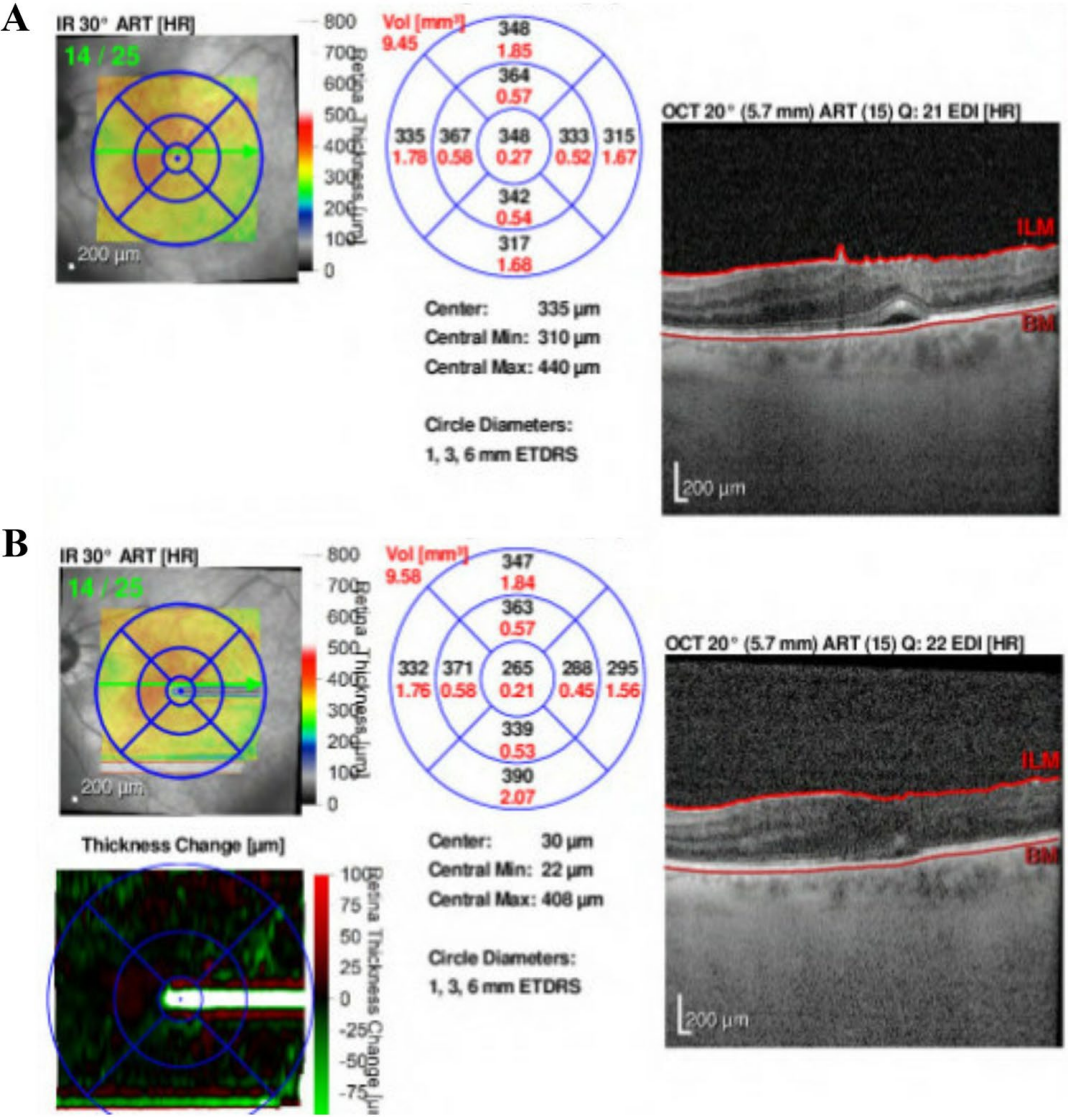
(**A**) Reference optical coherence tomography (OCT) from the visit prior to the fluorescein angiogram. (**B**) OCT from the same day as the fluorescein angiogram showing improved cystoid macular edema after injection of bevacizumab

**Fig. 5:**
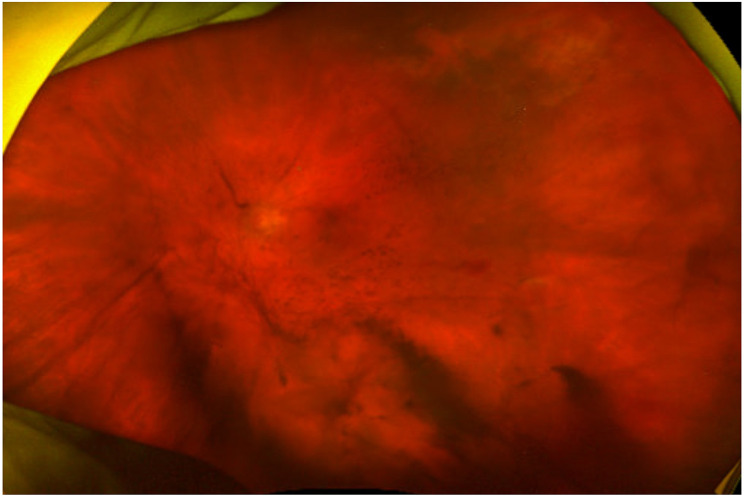
Latest widefield fundus photo of the left eye with persistent vitreous hemorrhage but no recurrence of the CMV retinitis

The CMV retinitis remained inactive; however, CME and vitreous hemorrhage persisted. Intravitreal or periocular corticosteroids were avoided due to concern for CMV reactivation in the setting of recent immune recovery uveitis. Thus, intravitreal bevacizumab (1.25 mg/0.05 mL) was administered, resulting in improvement of both CME and hemorrhage (Fig. 4A-B). Use of intravitreal bevacizumab for CME in this context represents off-label therapy. At the next visit, visual acuity improved to 20/125, with slow resolution of hemorrhage and CME and no recurrence of retinitis (Fig. 5). At a subsequent visit, the patient developed a new area of retinal whitening remote from the prior retinitis. Repeat anterior chamber paracentesis demonstrated negative aqueous PCR for CMV and other possible causative agents. The patient was evaluated by a vitreoretinal surgeon for consideration of pars plana vitrectomy. Given that retinal ischemia was the likely driver of the patient’s vitreous hemorrhage, pan-retinal photocoagulation is planned as prophylactic treatment to reduce the risk of recurrence and provide more durable ischemic control.

## Discussion and conclusions

CMV retinitis is most commonly seen in severely immunocompromised individuals, such as those with late-stage HIV or acquired immunodeficiency syndrome (AIDS), with reported prevalence ranging from 20 to 40% in this population [4, 11]. CMV retinitis has increasingly been recognized in patients receiving targeted immunomodulatory therapies, including JAK inhibitors. Although CMV reactivation can occur with JAK inhibitors, ischemic complications such as retinal ischemia and hemorrhage have not been well characterized in this population.

Although several cases of CMV retinitis in JAK inhibitor recipients appear in the broader literature, many occurred in individuals with hematologic malignancies, stem-cell transplantation, or concurrent chemotherapy, settings already associated with significant CMV risk. To maintain clinical relevance to our patient, we restricted our review to non-HIV, non-transplant, and non-malignancy patients without cytotoxic chemotherapy. Additional case descriptions exist, such as CMV retinitis following short-term tofacitinib exposure in patients concurrently receiving methotrexate or systemic corticosteroids, but these cases involved overlapping immunosuppressive therapies that made it difficult to attribute CMV infection to JAK inhibition alone and were therefore not included under our criteria [12]. Under these strict criteria, we have identified three published cases; two in patients receiving tofacitinib and one in a patient receiving upadacitinib [6–9]. None of these cases reported remote retinal ischemia or vitreous hemorrhage. Thus, while CMV retinitis is increasingly recognized in this population, ischemic sequelae appear to be uncommon and may be underrecognized.

Importantly, ischemic manifestations of CMV retinitis have been well documented in other immunosuppressed populations. Cho et al. described profound peripheral nonperfusion and neovascular complications in a stem-cell transplant recipient on daratumumab, illustrating that CMV-associated occlusive vasculitis can occur even without HIV infection [10]. However, such ischemic presentations have not previously been reported in the limited JAK inhibitor-associated cases. Our patient, therefore, broadens the clinical spectrum by demonstrating retinal ischemia remote from the site of retinitis and vitreous hemorrhage.

The anatomic dissociation between the original superotemporal retinitis and the temporal ischemic zone suggests an occlusive vasculitis rather than direct necrosis or simple extension of disease. Without this context, the ischemia could easily be misinterpreted as age-related microvascular disease without careful correlation to the patient’s earlier infection.

Mechanistically, JAK inhibitors impair antiviral immune surveillance by reducing JAK-STAT phosphorylation, decreasing interferon signaling, and down-regulating interferon-stimulated gene expression [13, 14]. Because CMV exposure is common in adults, reactivation typically occurs only when interferon-dependent immune control is weakened. In this case, treatment with upadacitinib with hydroxychloroquine likely created sufficient immunomodulation to permit CMV reactivation and development of retinitis, despite the absence of profound systemic immune deficiency. Although the patient was also receiving long-term hydroxychloroquine, this medication is an immune-modifying agent rather than an immunosuppressant. Its mechanism of action does not impair interferon signaling or antiviral immune surveillance [15]. Therefore, upadacitinib remains the most plausible contributor to impaired antiviral control that permitted CMV reactivation in this case.

Although our patient had several systemic vascular risk factors, including advanced age, chronic kidney disease, clonal cytopenia, and hypertension, these were present bilaterally, yet ischemia occurred only in the eye affected by CMV retinitis. The fellow eye remained perfused without hemorrhage, suggesting that these comorbidities alone were insufficient to explain the unilateral ischemic changes. Alternative vascular etiologies such as idiopathic retinal vasculitis, aneurysms, and neuroretinitis (IRVAN) were also considered. However, the focal aneurysmal changes on fluorescein angiography were confined to the temporal ischemic zone and were absent in the fellow eye, which is atypical for IRVAN. The unilateral involvement and the anatomical separation between the CMV retinitis and the subsequent ischemic region, therefore, support CMV-associated occlusive vasculitis as the primary precipitating factor.

Management required careful coordination between ophthalmology and rheumatology to balance infection control with underlying autoimmune treatment. Ischemia was the likely driver of vitreous hemorrhage; therefore, pan-retinal photocoagulation is planned to reduce recurrent bleeding. The patient later developed a new area of retinal whitening; repeat aqueous PCR was negative, and she was evaluated for a possible pars plana vitrectomy, reflecting the chronic and unpredictable nature of post-infectious sequelae.

This case expands on the literature on CMV retinitis associated with JAK inhibition and demonstrates that ischemic complications can occur even in moderately immunosuppressed individuals. As JAK inhibitors become more widely prescribed, clinicians should maintain heightened vigilance for CMV retinitis and its delayed vascular consequences, including ischemia, neovascularization, and hemorrhage, even after apparent stabilization of the primary infection. Additional reports will be important for defining the clinical spectrum and informing monitoring strategies in this emerging patient population.

## Data Availability

The data that support the findings of this study are available on request from the corresponding author. The data are not publicly available due to privacy or ethical restrictions.

## Abbreviations

AIDS: Acquired immunodeficiency syndrome
Anti-VEGF: Anti-vascular endothelial growth factor
CME: Cystoid macular edema
CMV: Cytomegalovirus
HIV: Human immunodeficiency virus
IRU: Immune recovery uveitis
JAK: Janus kinase
OCT: Optical coherence tomography
PCR: Polymerase chain reaction
RA: Rheumatoid arthritis
